# Progress in Surgery

**Published:** 1898-11-26

**Authors:** 


### Progress in Surgery.

### SURGERY OF THE VERMIFORM APPENDIX.

Pond[1] divides appendicitis into the following forms: (1) Simple inflammation of mucous membrane of the appendix, resulting in resolution—so-called catarrhal appendicitis. (2) The same, with or without the formation of calculi, either resulting in resolution or going on to gangrene and perforation. (3) A class of cases which rapidly within twelve or twenty-four hours goes on to gangrene or perforation with peritonitis or septicæmia. (4) A type characterised by rapid lymphatic poisoning, in which neither gangrene, nor perforation, nor peritonitis is present. (5) Appendicitis abscesses. (6) Chronic appendicitis. The author takes the extreme view as regards operation, which, he says, should be performed as soon as the diagnosis is made, no matter how mild the case may be, unless the patient is in severe shock following sudden perforation. In such cases first overcome the shock. In bad cases of appendicitis, when the abdomen is filled with pus and the intestines are paralysed by over distension, morphine, and infection, an opening into the intestines to promote intestinal drainage will be of benefit. Bryant[2] calls attention to a class of cases of chronic appendicitis characterised by abundant fibrinous exudation, and makes the following suggestions as an outcome of his study of these cases: (1) That an enormous deposit of lymph may be the only product of some forms of appendicitis. (2) That though repeated attacks occur, suppuration is not a common complication of this condition. (3) That free incision of the mass, attended by light packing with gauze, is followed by rapid absorption of the induration, and often by cure. (4) That suppuration may occur; therefore the appendix should be at once removed if possible; if not, as soon thereafter as possible. (5) That chronic inflammation of the appendix, with fluctuations of a mild degree, provokes a response of apparently similar nature in the surrounding fibrinous mass. (6) That fibrinous induration, due to chronic appendicitis, may be mistaken for sarcoma of the cæcum. (7) That the influence of malaria may stimulate the fibrinous variety of chronic appendicitis, and that possibly the symptoms may be dispelled by the use of quinine. Hare[3] says, that although operation is not necessary in all cases of appendicitis, there are cases which are so fulminating in character that an hour's delay is dangerous, and he believes that in cases with early fixation of the body and abdominal muscles, and without much temperature disturbance, operation is more necessary than in those in which the pain is greater, more continuous, and more severe on pressure. Halliday[4] gives a record of 100 consecutive cases of appendicitis, divided into three classes: 1. Simple appendicitis without suppuration, 64 cases. 2. Appendicitis with abscess formation, 22 cases. 3. Acute perforative appendicitis, with general purulent peritonitis, 14 cases. Of the 64 cases in class 1 the appendix was removed during an interval in 25, and one case was fatal, giving a mortality of 1·6 per cent. Of the 22 cases in class 2, 17 recovered and five died, giving a mortality of 23 per cent. In one case the abscess recurred after some months, and in two the appendix had to be removed subsequently for recurrent simple attacks. The 14 cases in class 3 were all fatal. The most noticeable feature about this class are, that out of the 14 cases 10 occurred between the ages of 10 and 20 years, and in 10 cases the attack was the first. These patients were generally admitted *in extremis*, with a history of from three to eight days' illness. The abdomen was opened in eight cases, and in the others the diagnosis was verified, or made at the necropsy. On the subject of fulminating appendicular inflammation Crutcher[5] quotes many eminent authorities to show that there is a consensus of opinion that it is impossible to foretell perforation, while, as a rule, when fulminating appendicitis has arrived at the stage when it can be diagnosticated as such, it is worse than useless to operate. He thus summarises his own conclusions: (1) If pain is violent, sudden, and persistent, it indicates probable seriousness of the attack, but nothing as to the natural defences for limiting infection. On the other hand, absence of pain is undecisive between gangrene and resolution. (2) While a rapid pulse and high temperature favour the destructive process, their absence affords no assurance of recovery. Gangrenous appendicitis may exist in the presence of normal temperature. (3) The presence of shock, if undoubted, is a very grave symptom, generally indicating perforation. On the other hand, the most deadly attacks often occur without it. (4) If tenderness is persistent and highly developed, it generally indicates destructive inflammation, but gives no clue concerning the limitation of infection. (5) The facial expression is of material value before the development of grave conditions. (6) Perforation can no more be foretold than the perforation of the intestine in typhoid fever, or the rupture of an aneurism. Ferguson[6] says that taking all the cases of appendicitis, it is probable that from 80 to 90 per cent. will recover from the first attack without perforation or suppuration. Of those who do not so recover, from 80 to 90 per cent. will have a circumscribed collection of pus, and will be relieved by an exceedingly simple and fairly safe operatic procedure. The very small percentage left will have more serious conditions, including the rapidly generalised septic peritonitis. Of these cases a very large per cent. will perish, but it can scarcely be desirable to operate on all cases of appendicitis in order to save these few cases, for the mortality of the operation itself is from 5 to 10 per cent. The author therefore concludes that in all cases of appendicitis during the first attack operation should not be performed unless suppuration or diffuse peritonitis requires it. In lapsing or recurrent cases, in which it is probable that distortion or other permanent injury to the appendix exists, operation should be performed. Bemays[7] believes that the safest plan of management of all cases of appendicitis is the immediate removal of the appendix and the drainage of the affected cavities. This plan of treatment will be followed by about 98 per cent. of recoveries. The expectant plan in a large percentage of the cases will lead to a late surgical operation, and in about 10 per cent. of these cases will lead to death. Morris[8] says it has been determined from statistics of many hundreds of operations, at the hands of several American surgeons, that the surgical death rate of appendicitis is less than 1 per cent. in the class of cases in which infection is limited to the confines of the appendix proper. This includes primary infection cases before it has spread beyond the appendix and the so-called "internal cases." It has also been determined that proper surgical operation in cases of widespread infection outside of the confines of the appendix proper, can lower the death-rate in this class of cases to about 5 per cent., operation being performed upon all patients who are still living when the surgeon arrives. The death-rate of cases treated medically is not likely to be less than 25 per cent. eventually, although it is believed that the death-rate in first attacks is not much above 10 per cent. Southam[9] quotes cases to show that some cases of perityphlitic abscess are undoubtedly of cæcal origin; and Andrews[10] records a successful case of operation for a perforating appendicitis in which general peritonitis had occurred.

[1] Med, Rec., April 23, 1898, p. 583. [2] Med. Chron., April, 1898, p. 43. [3] Ibid., p. 48. [4] Brit. Med. Jour., May 7. 1898, p. 1195. [5] New York Med. Jour., May 28, 1898, p. 766. [6] Ibid., Mar. 26, 1898, p. 425. [7] Med. Rec., April 2. 1898, p. 478. [8] Ibid., May 21, 1898, p. 728. [9] Brit. Med. Jour., April 30, 1898, p. 1130. [10] Lancet, May 28, 1898, p. 1464.

